# Parenthood and Self-Reported Depression, Anxiety, and Life Satisfaction in the COVID-19 Pandemic in the UK: An Examination of Differences by Age of Children and Level of Social Support

**DOI:** 10.3390/ijerph22111664

**Published:** 2025-11-02

**Authors:** Hannah Jones, Marie Houghton, Jorge Gato, Fiona Tasker

**Affiliations:** 1 School of Psychological Sciences, Birkbeck, University of London, London WC1E 7HX, UK; h.jones13@exeter.ac.uk (H.J.);; 2 Faculty of Health and Life Sciences, University of Exeter, Exeter EX1 2LU, UK; 3 Faculty of Psychology and Education Sciences, University of Porto, Faculty of Psychology and Education Sciences, University of Porto, Rua Alfredo Allen, 4200-135 Porto, Portugal; jorgegato@fpce.up.pt

**Keywords:** mental health, wellbeing, parenthood, COVID, pandemic, stress process model, social support

## Abstract

The COVID-19 pandemic had a marked detrimental effect on the mental health of the UK population. Parents with dependent children were deemed vulnerable but research on parental mental health in this period neglected to examine a child’s age together with the presence of social support. To inform potential mental health support strategies this study investigated whether the pandemic was associated with different levels of psychological wellbeing for parents with youngest children of varying ages, relative to socio-demographic factors and social support levels. From November 2020 to April 2021 n = 915 UK adults completed an online survey measuring self-reported symptoms of depression, anxiety and stress, satisfaction with life, social support and socio-demographic characteristics. Results provide some evidence of better psychological wellbeing for parents with younger children (aged 0 to 5 years) than older children. Overall, social support was a key factor in mitigating depression, anxiety, and stress scores for parents of dependent-aged children. Findings provide new evidence supporting Pearlin’s Stress Process Model, highlighting the importance of social support to parents under pandemic-related pressures. These findings indicate that one way of safeguarding parents vulnerable to poor mental health could be by increasing social support to parents via formal and informal support services within school communities.

## 1. Introduction

The coronavirus (COVID-19) pandemic and the measures put in place by Governments to contain it, affected lives around the world in profound and unprecedented ways. In the UK, from the imposition of the first nationwide ‘lockdown’ on 26 March 2020, people were told to work from home where possible, non-essential shops were closed, travel was restricted, and contact with people outside the household was limited. While these measures helped to slow the spread of the virus, the psychological effects of this sudden imposition of social restrictions added to the psychological impact of the pandemic and became an increasingly voiced public concern [1,2]. The UK Office for National Statistics (UK-ONS) estimated that the proportion of adults experiencing some form of depression (defined as experiencing moderate to severe symptoms of sadness, helplessness, and listlessness, indicated by a score of 10 or more on the eight-item Public Health Questionnaire (PHQ8)) more than doubled, from 10% pre-pandemic to 21% by March 2021 [3], and almost half of the population experienced high levels of anxiety (defined as an anxiety rating of 6 or more out of 10 on the ONS4 Anxiety question) at some point during the pandemic [4,5,6]. Nonetheless, behind the overall increase in mental health problems, variability was noted [6,7,8]. Furthermore, some evidence suggested that although elevated depression and anxiety levels remained among high-risk groups, life satisfaction began to return to pre-pandemic levels during the second year of the pandemic [9].

One group who appeared to be particularly vulnerable to negative mental health impacts from the pandemic were parents caring for dependent children (‘dependent child’ is defined by the Office of National Statistics (ONS) as a person aged 0 to 15 years in a household or a person aged 16 to 18 years who is in full-time education and lives in a family with their parent(s) or grandparent(s)). Several studies of adult mental health during the early stages of the pandemic in the UK [3,6,10] and elsewhere [11] highlighted parents as one of the groups experiencing a particularly marked decline in psychological wellbeing. Particular concern focused on women’s mental health in this regard [12], largely as a result of the greater proportion of unpaid childcare undertaken by mothers pre-pandemic [13,14], and the intensification of this inequality during the pandemic [15] with the closure of schools and formal childcare facilities. As noted by Geprägs et al. [9], this was particularly acute during the first year of the pandemic—the lifting of social restrictions during the second year, with the associated re-opening of schools and childcare facilities, eased this additional childcare burden.

Nevertheless, there has been limited and inconsistent evidence concerning the psychological effects of the pandemic and social restriction periods on parents of dependent children at different developmental stages. One UK study indicated that a key group at risk of deteriorating mental health in the pandemic was parents with a youngest child aged 0 to 5 years [6]. However, another UK study emphasised that parents of school-age children (compulsory school age in the UK is from the term after a child’s fifth birthday until the end of the academic year in which they turn 16) were at particular risk, mainly because they were more affected by the enforced school closures [10].

Furthermore, the vast majority of studies of parental mental health in the pandemic have not considered how positive gains in parental psychological wellbeing might offset parental strain brought about by the pandemic. A notable exception is a study by Nomaguchi et al. [16], which found evidence of a mental health advantage for women with children living at home during the pandemic in the U.S. Although Nomaguchi and colleagues did not examine whether outcomes differed by age of children, research prior to the pandemic indicated gains in wellbeing associated with being a parent of a child under five years, whereas life satisfaction was lower for parents of older children [17].

The Stress Process Model (SPM) [18] can offer a theoretical framework for understanding why different individuals and groups, such as parents of dependent children, may experience different levels of stress from an overall ‘stressor’ such as the COVID-19 pandemic and associated restrictions. In Pearlin’s SPM, stress is contextualised as a process involving three major components: manifestations, stressors, and mediators. Manifestations consist of the outcomes through which stress is revealed, namely the impact upon mental and physical wellbeing. Stressors are seen as the sources of stress from either discrete life events or ongoing problems; here the pandemic has been interpreted as presenting both types of challenges. Mediators are defined as the personal and social resources through which an individual may attempt to alleviate the impact of a stressor, including the social support available from an individual’s social network [18]. Thus, working from the basis of Pearlin et al.’s model, we predicted that when experiencing stress from the pandemic and the associated social restrictions individuals who report greater levels of social support will also record higher levels of wellbeing. Previous research has highlighted social support as a stress mediator in determining wellbeing outcomes during the pandemic [5,19,20,21], and research in diverse settings has shown the importance of considering the role of social support when adults are experiencing stress [22]. Nevertheless, social support has yet to be examined specifically in relation to parental wellbeing—as defined by the American Psychological Association as a ‘state of happiness and contentment, with low levels of distress, overall good physical and mental health and outlook, or good quality of life’ [23]—during the pandemic as we consider it here in the current study.

The present study aimed to contribute to these research gaps by analysing (i) the association of being a parent of pre-school or school-aged children with both negative and positive measures of wellbeing, over and above the relative contribution of demographic factors, during the second wave of the pandemic, and (ii) the ameliorative effects of social support on parental wellbeing. Considering these research aims we tested the following hypotheses, with reference to the second wave of the pandemic:

**Hypothesis 1.**  *Parents with their youngest child aged 15 or under will have higher levels of depression, anxiety, and stress, and lower levels of satisfaction with life than those participants who were not parents or who had a child aged 16 years and older.*

**Hypothesis 2.**  *Parents with a youngest child aged 6 to 15 years will have higher levels of depression, anxiety, and stress, and lower levels of satisfaction with life than those with a youngest child aged 0 to 5 years.*

**Hypothesis 3.**  *Mothers will have higher levels of depression, anxiety, and stress, and lower levels of satisfaction with life than fathers.*

**Hypothesis 4.**  *Demographic factors (age, gender, health, and employment status) will be associated with different levels of depression, anxiety, and stress, and satisfaction with life, independent of parenthood status.*

**Hypothesis 5.**  *Higher levels of perceived social support will be associated with lower levels of depression, anxiety, and stress, and higher levels of satisfaction with life, independent of age of youngest child.*

## 2. Materials and Methods

### 2.1. Participants

The survey sample comprised 915 cisgender heterosexual participants resident in the UK (76.5% female, 23.5% male). Sample characteristics are summarised in Table 1. The mean age was 42.6 years (*SD* = 12.9). While the majority (72.1%) did not have a physical or mental health condition or disability, 27.9% did. Most were employed full- or part-time (68.9%), compared to 10.6% who were not employed and 6% who were students. Parents with a youngest child aged 0 to 5 made up 10.2% of the sample, and parents with a youngest child aged 6 to 15 comprised 11.9%.

### 2.2. Recruitment

This study was based on information collected via an online survey of adults residing in the UK between 18 November 2020 and 14 April 2021. Recruitment methods included: advertisements on Google and Facebook, circulation of flyers in public places (e.g., posters on community noticeboards), and placement of messages with study details on relevant online groups such as parenting forums. Participants were asked to complete the survey anonymously if they were aged 18–60 years and resident in the UK. All participants were shown information about the content and purpose of the study and confirmed their informed consent in writing if they wanted to take part. No paid incentive was offered to participants. Ethical approval was granted by an Institutional Review Board.

### 2.3. Measures

The survey was anonymously accessed by participants online through a secure link hosted on SurveyMonkey. In addition to demographic information gathered by standard UK census style questions, we asked whether participants were a parent or stepparent to a dependent child (aged under 16 years old), and whether the child resided with them some or all the time. As described below, participants were asked to indicate the extent of social support they had received using the Multidimensional Scale of Perceived Social Support and to record their psychological wellbeing using two established tests: the Satisfaction with Life Scale and the Depression, Anxiety, and Stress Scale.

The three-item (seven-point Likert-type rating scale) Satisfaction with Life Scale (SWLS-3) [24], based upon Diener et al.’s [25] established scale, was used to capture participants’ global appraisal of their lives, relative to their hopes and expectations, with increasing scale scores equating to greater satisfaction. The current study found good internal consistency for the SWLS-3, with Cronbach α = 0.93. Diener’s original Satisfaction with Life Scale was designed to give a score of the participant’s overall cognitive assessment across all domains of their life, not an affective rating of success in any specific area, such as work or family. The original 10-item SWLS was created out of a pool of 48 items by selecting the 10 items that best distinguished global life satisfaction from either positive or negative affect [25]. The SWLS scale has an established track record of reliability and validity in a variety of study contexts [26].

Participants’ levels of depression, anxiety, and stress were measured using the 21-item Depression, Anxiety, and Stress Scale (DASS-21) [27]. Participants ranked 21 statements regarding their distress levels in the last week, from zero (did not apply to them at all) to three (applied to them very much). The current study found good internal consistency for all three individual sub-scales: Depression Cronbach α = 0.93, Anxiety Cronbach α = 0.85, and Stress Cronbach α = 0.89.

Perceived level of social support was measured using the Multidimensional Scale of Perceived Social Support (MSPSS) [28]. The scale comprised twelve questions that capture individuals’ perceptions of the total amount of support received from their family, friends, and a significant other (partner or closest friend). Respondents chose an answer from a 7-item Likert scale to denote their level of agreement with a series of statements, with higher scale scores indicating greater perceived social support. The current study found good internal consistency for the MSPSS total score, with Cronbach α = 0.94.

### 2.4. Analyses

Initially information from the full survey cohort was explored using ANOVAs to establish: (i) whether men and women who parented dependent children under 16 years old had higher levels of depression, anxiety, and stress, and lower levels of satisfaction with life than did either those who parented a child of 16 or over or who were not a parent or stepparent; (ii) whether men and women with a youngest child aged 6 to 15 years had higher levels of depression, anxiety, and stress, and lower levels of satisfaction with life than those with a youngest child aged 0 to 5 years; and (iii) whether mothers with a dependent child under 16 years had higher levels of depression, anxiety, and stress, and lower levels of satisfaction with life, compared with fathers (testing Hypotheses 1 to 3).

Subsequently Hierarchical Multiple Regression (HMR) models were used to look specifically at the group of parents with a youngest child aged under 16, to examine the impact of having a youngest child aged 0 to 5 years, compared to a youngest child aged 6 to 15 years, alongside differences in levels of perceived social support and existing demographic contributors to wellbeing during the UK’s second wave of COVID-19 infections (testing Hypotheses 2 to 5). The five independent, predictor variables and associated measures were as follows and grouped into three blocks in the HMR models: block 1 consisting of demographic characteristics (age, presence of disability or health condition, and employment status); block 2 entering parent status with parents of children under 16 years old grouped into two categories (i) parents with their youngest child aged 0 to 5, and (ii) parents with their youngest child aged 6 to 15; and block 3 entering level of perceived social support. The two dependent outcome variables were: level of satisfaction with life and the combined total of depression, anxiety, and stress scores reported.

Prior to analysis, preliminary data checks were carried out to assess the suitability of the sample. The sample size of 158 parents with a child under 16 years old was considered adequate for the inclusion of five independent variables in the HMR analysis. Outliers were checked and found in one of the independent variables (DASS score), but these were not removed as normal distribution cannot be assumed for psychological variables and did not affect the analyses given the sample size [29]. Ahead of the HMR analysis, assessment of scatterplots demonstrated the required linearity of relationships. Tests of collinearity (VIF and Tolerance) showed that there was no perfect linear relationship between predictor variables. Finally, Durbin-Watson statistics were calculated, establishing that there was no autocorrelation between residuals, and that errors were independent.

## 3. Results

### 3.1. Differences in Average Scores of Wellbeing Indicators by Parent Status and Gender

Parenthood status was broken down into categories to enable a comparison between those with a youngest child aged 0 to 5 years versus those with a youngest child aged 6 to 15 years. The analysis below first considers differences in wellbeing outcomes between men and women with a youngest child aged 0 to 5 years, 6 to 15 years, and 16 or older; and those who were not parents (see summary of average scores for depression, anxiety and stress, and satisfaction with life in Table 2).

To establish whether there were statistically significant associations between parenthood, gender, and wellbeing in the pandemic, a two-way independent-groups analysis of variance (ANOVA) was conducted on the two wellbeing outcome variables (level of satisfaction with life and level of depression, anxiety, and stress), with age included as a covariate. Here there was no significant main effect of parental status (parent of youngest child aged 0 to 5 years; 6 to 15 years; 16 or older; non-parents) for satisfaction with life, F(3, 587) = 2.25, *p* = 0.082. Similarly, there was no significant main effect for gender, F(1, 587) = 2.57, *p* = 0.110, or interaction between parental status and gender, F(3, 587) = 1.88, *p* = 0.131.

However, a significant main effect was found for parental status for levels of depression, anxiety, and stress, F(3, 541) = 3.64, *p* = 0.013. Pairwise comparison of parental status groups revealed significantly higher levels of depression, anxiety, and stress for those with a youngest child aged 6 to 15, compared to those with a youngest child aged 0 to 5 (*p* = 0.018). Those with a youngest child aged 16 or older were also found to have higher levels of depression, anxiety, and stress, compared to those with a youngest child aged 0 to 5 (*p* = 0.043). No significant differences were found between levels of depression, anxiety, and stress for those who were not parents compared to parents of any age child.

### 3.2. Associations Between Demographic Characteristics, Parenthood Status, Perceived Level of Social Support, and Wellbeing Indicators

The associations between wellbeing outcome measures and parenthood of pre-school and school-age dependent children were further investigated using HMR models, testing the relative predictive power of these characteristics against demographics and level of social support. To enable this staged exploration, background demographics (age, disability/health condition, and employment status) were entered first (gender was excluded from HMR analysis as it was not found to be significant in the prior ANOVA analysis), then parenthood status (split by age of youngest child 0 to 5, and 6 to 15 years) was added to assess the additional explanatory power that this information gave. The third step allowed the impact on wellbeing of social support to be independently evaluated. Two HMRs were conducted, one for each of the wellbeing indicators measured.

#### 3.2.1. Satisfaction with Life Score

Table 3 shows the results of the regression model for Satisfaction with Life Score (SWLS). The first model, containing only demographic information, was overall significant (F(3, 97) = 3.014, *p* = 0.034), although it only explained 8.5% of the variance in SWLS. Living with a disability or pre-existing health condition (B = −0.274, *p* = 0.008) was negatively associated with SWLS. There was no significant relationship between SWLS and employment status or age in Model 1. The addition of information about age of youngest child in Model 2 did not improve the overall predictive power of the model (F(1, 96) = 0.792, *p* = 0.376). Thus, having a youngest child of different ages (0 to 5, compared to 6 to 15 years) was not significantly associated with SWLS, when demographic information was controlled for. The inclusion of social support in Model 3 significantly improved the fit of the overall model (F(1, 95) = 10.250, *p* = 0.002) and predicted an additional 8.8% of the variance of SWLS. A higher level of perceived social support was associated with better SWLS (B = 0.336, *p* = 0.002). No other characteristic (having a disability or health condition, age, employment, or age of youngest child) was found to be statistically significant once level of social support was added to the model.

#### 3.2.2. Depression, Anxiety, and Stress

Table 4 shows the results of the regression model for depression, anxiety, and stress (measured using DASS-21). Overall, the first model, containing only demographic characteristics, was statistically significant (F(3, 92) = 11.068, *p* < 0.001), and predicted 26.5% of the variance in depression, anxiety, and stress (DAS). Again, having a disability or health condition (B = 0.459, *p* < 0.001) was associated with higher levels of DAS and remained statistically significant in subsequent models. The addition of the age of the youngest child in the second model improved the proportion of the variance of DAS explained, with an increase of 6.5% (F(1, 91) = 8.859, *p* = 0.004). Being a parent with a youngest child aged 0 to 5 years was related to lower levels of DAS (B = 0.305, *p* = 0.004), as was being older (B = −0.313, *p* = 0.004). The inclusion of perceived level of social support (Model 3) added further predictive power, explaining an additional 4.3% of DAS variance (F(1, 90) = 6.188, *p* = 0.015). Greater levels of social support were related to lower levels of DAS (B = −0.243, *p* = 0.015). All characteristics which were significant in the previous models remained so, after the inclusion of perceived social support in model 3.

## 4. Discussion

Using Pearlin’s Stress Process Model [18] as a theoretical lens, our UK survey conducted during the second wave of the COVID-19 pandemic (November 2020–April 2021) examined, firstly, whether parents of dependent children experienced poorer mental wellbeing compared to that of adults who were not responsible for children aged less than 16 years old and, secondly, whether those with a youngest child of school-age were particularly adversely affected. When considering the age of the children being cared for and the associated parental strain and reward incurred, we examined whether wellbeing effects were different for mothers and fathers and whether differences in the level of social support modified parental wellbeing scores.

Simply examining parenthood status provided some evidence that parents who had a youngest child aged 0 to 5 years fared better than those with school-age children in the pandemic, in terms of lower levels of depression, anxiety, and stress. A similar pattern favouring parents with younger children was found when comparing parents with their youngest child aged 0 to 5 years and parents with those whose youngest child was 16 years old or more. However, no differences in depression, anxiety, and stress were detected when comparing those who were not parents with any of the groups of parents.

Further analysis of those parents with dependent children under the age of 16—comparing those with a youngest child aged 0 to 5 with those aged 6 to 15 years—revealed that having a youngest child aged 6 to 15 remained significantly associated with higher levels of depression, anxiety, and stress, once perceived level of social support and other demographic characteristics (notably participants’ health and age) were considered. These results are in line with the findings of Nomaguchi [17], who found that parents whose children were aged under five years had better mental health than parents of older children, which was found to be largely driven by better parent–child relationship quality. Nomaguchi’s study was undertaken pre-pandemic; therefore, findings from the present study provide some evidence that this overall pattern of positive wellbeing among parents of young children remained throughout the pandemic.

Irrespective of the child’s age, having a disability or a physical or mental health condition, and being younger, were reliably associated with higher levels of depression, anxiety, and stress. It was consistently found that higher levels of perceived social support were related to better wellbeing scores across both measures of depression, anxiety and stress, and satisfaction with life, mirroring findings from the previous literature [5,19,20,21].

No evidence was found in the present study to support the hypothesis that mothers would experience poorer depression, anxiety and stress, or satisfaction with life outcomes than fathers. Thus, in the second wave of the pandemic in the UK, our findings indicated no difference in maternal or paternal wellbeing, unlike the findings pointing to the specific wellbeing deficit experienced by mothers in the UK during the first pandemic wave [10]. Here the findings reported in our present study replicate those of Geprägs et al. [9] who found that gender had no significant association with quality of life during the second wave of the pandemic in Germany.

The most compelling explanation for the difference between the present study’s results and those of Geprägs et al. [9] compared to other studies is the difference in data collection dates. The survey for the current study was carried out between November 2020 and April 2021, during the peak of the second wave of COVID cases in the UK. The majority of these other studies [5,6,10] took place during the first lockdown. Crucially, schools were closed for the April–June 2020 period during the first UK lockdown. In contrast, the time span of the current survey included a mixture of lockdown and non-lockdown periods, with schools fully closed only some of the time (5 January to 15 March). From research such as that by Andrew et al. [30], a key factor behind the steeper decline in maternal, as compared with paternal, mental health was shouldering much of the burden of increased responsibility for home-schooling and childcare during school closures. This added pressure of home-schooling was not consistently present during the data collection period of the current study, which may go some way to explaining the discrepancy between our findings of no gender differences and those from other studies [5,6,10] where the research data were collected in the first wave of the pandemic.

Overall findings from the current study have indicated consistent support for Pearlin’s [18] theory regarding the relationship of social support with stress and mental wellbeing for parents of dependent pre-school and school-aged children. Higher levels of perceived social support were found to significantly predict higher levels of satisfaction with life and lower levels of depression, anxiety, and stress—over and above the age of the youngest child parented and other demographic characteristics. This is an important contribution to the existing literature on the mental health effects of the pandemic. While other studies [19,20,21] have highlighted the importance of social support to wellbeing in the pandemic, none to date (to the authors’ best knowledge) have demonstrated the importance of social support in offsetting known pandemic mental health risk factors, such as disability, age, gender, and employment status, alongside parenthood of children of different age groups.

### Strengths and Limitations of the Study

The present study has several key strengths that set it apart from existing studies in the field. Firstly, this study incorporated the important insights of Pearlin’s [18] theoretical Stress Process Model to propose that social support is vital to consider when assessing the impact of stress processes on wellbeing and findings from our study justify this decision. Other research that includes consideration of social support does not consider it relative to the role of parenthood alongside other key demographic risk factors for mental health [19,20,21]. Secondly, the role of parenthood was examined in conjunction with the age of children. This is an important differentiator in the wellbeing outcomes of parents, as evidenced in Nomaguchi’s [17] pre-pandemic research, yet the age of the parent’s child is missing information in many research studies considering risk factors for parental mental health in the pandemic (Pierce et al. [6] being a notable exception). In addition, the current study considered positive as well as negative psychological wellbeing, allowing a more nuanced analysis of changes in mental health.

Notwithstanding the strengths of the current study, there were also some limitations. As in many psychological surveys of this type, our study relied on self-report measures of psychological wellbeing, and there is no triangulation of findings with behavioural data or independent assessments. There is potential for perceptions of social support to be affected by levels of depression—while this interaction cannot be controlled, it is important to acknowledge it. Another limitation concerned the cross-sectional nature of the survey, meaning that we were unable to track change in mental health over the course of the pandemic, unlike findings by Pierce et al. [6] and Blanden et al. [10].

Furthermore, the sample recruited was not a random probability sample, which would have provided the optimum conditions for validity and generalizability of results [31]. Instead, our participants were invited to participate through advertisements placed on Facebook and Instagram, as well as via snowball sampling. Adverts were also placed on parenting forums, which could have introduced bias to the sample (for example, if parents who participated in the survey were those particularly concerned with education and school attendance).

We examined whether the age profile of our sample differed from that studied by Pierce et al. [6], and the UK-ONS samples [3,5], which all approximated to the mean age of the UK population (41.2 years). The mean age of the current study was 42.6 years and therefore similar to the UK national average. In addition, the proportion of those in our sample in the 18 to 24 age bracket, and thus most at risk of poor mental health, was again comparable to the proportion in the general UK population (8.8% compared to 8%). We also considered the gender make-up of this study’s sample. The ratio of female to male participants was over 3:1, which could mean that the sub-sample of males was too small to pick up meaningful differences in mental health outcomes. However, the overall sample size was large (n = 915), meaning that even the smaller male sub-sample (n = 215) was comfortably over the minimum requirement. Nevertheless, as the current study did not use a stratified sampling frame, we cannot completely rule out sampling effects.

## 5. Conclusions

The present study has contributed to understanding how parenting children of different ages impacts mental wellbeing in the UK COVID-19 pandemic. In line with findings from studies during the first wave of the pandemic, such as Blanden et al. [10], we found evidence of an association between being a parent of a school-aged youngest child and higher levels of depression, anxiety, and stress, compared to parents with a youngest child of 5 years or less. However, findings indicated that those with older children (measured as a youngest child aged 16 years and above) also showed a similar pattern of higher levels of depression, anxiety, and stress. No evidence of difference was found for non-parents.

It was also particularly notable that higher levels of social support were consistently related to better mental wellbeing outcomes, irrespective of demographic characteristics and the age of the youngest child. These findings replicate and build upon other pandemic-focused research on this topic [19,20,21]. In particular, the findings of the current study provide new evidence supporting Pearlin’s [18] Stress Process Model, indicating that one practical way of safeguarding those vulnerable to poor mental health would be through increasing social support to parents via early years or school community formal and informal support services. These findings are prescient as we pass the five-year anniversary of the COVID pandemic, with attention focused on the lessons society could learn from the experiences of this time.

## Figures and Tables

**Table 1:** Demographic characteristics of sample.

Demographic Characteristics	n (%)
**Gender**	
Female	700 (76.5)
Male	215 (23.5)
**Ethnicity**	
White British	763 (85)
White Non-British	63 (7.0)
Mixed	18 (2.0)
Asian	27 (3.0)
Black	17 (1.9)
Other	10 (1.1)
**Employment status**	
Employed—Full-time	400 (47.4)
Employed—Part-time	181 (21.5)
Not employed	89 (10.6)
Student	51 (6.0)
Other	122 (14.5)
**Health condition or disability**	
No	658 (72.1)
Yes	255 (27.9)
**Parent status**	
Parent with youngest child aged 0–5 years	73 (10.2)
Parent with youngest child aged 6–15 years	85 (11.9)
Parent with a youngest child aged 16 years or older	185 (25.9)
Not a parent	370 (51.9)

**Table 2:** Wellbeing outcome mean scores and standard deviation—gender and parenthood status.

	Gender	Parent with Youngest 0–5 years Mean (*SD*)	Parent with Youngest 6–15 years Mean (*SD*)	Parent with Youngest 16 years or Older Mean (*SD*)	Not a Parent Mean (*SD*)
Satisfaction with life (SWLS)	Men	12.44 (5.70)	8.64 (6.41)	11.29 (5.72)	10.00 (5.54)
	Women	12.93 (4.45)	11.33 (5.14)	10.41 (5.29)	11.78 (4.85)
Depression, anxiety, and stress (DASS)	Men	14.07 (15.84)	24.73 (16.08)	16.77 (15.66)	20.38 (15.16)
	Women	18.00 (11.95)	21.13 (13.43)	19.18 (13.86)	20.06 (14.16)

**Table 3:** Hierarchical regression: association of the predictor variables and Satisfaction With Life Score (SWLS).

	Model 1			Model 2			Model 3		
	B	SE	β	B	SE	β	B	SE	β
Disabled or health condition ^a^	−3.098	1.143	−2.74 **	−2.907	1.164	−0.258 *	−1.825	1.162	−0.162
Employment status ^b^	0.637	1.570	0.040	0.561	1.574	0.035	−0.693	1.553	−0.043
Age	0.17	0.061	0.029	0.051	0.072	0.084	0.059	0.069	0.096
Parent with youngest child 0–5 vs. 6–15 years				−1.052	1.183	−0.102	−0.291	1.154	−0.028
Social support							0.104	0.032	0.336 **
R^2^	0.085			0.093			0.181		
Adjusted R^2^	0.057			0.055			0.138		
R^2^ change	0.085			0.007			0.088		
F for R^2^ change	3.014 *			0.792			10.250 **		

* *p* < 0.05; ** *p* < 0.01. ^a^ Disability (0 = no disability or health condition, 1 = has disability or health condition); ^b^ Employment status (0 = not employed, 1 = employed (full- or part-time).

**Table 4:** Hierarchical regression: association of the predictor variables and total depression, anxiety, and stress score (from DASS-21).

	Model 1			Model 2			Model 3		
	B	SE	β	B	SE	β	B	SE	β
Disabled or health condition ^a^	13.705	2.766	0.459 ***	11.938	2.721	0.400 ***	10.146	2.742	0.340 ***
Employment status ^b^	−1.207	3.726	−0.029	−0.778	3.579	−0.019	1.543	3.604	0.038
Age	−0.234	0.148	−0.144	−0.508	0.170	−0.313 **	−0.532	0.165	−0.328 **
Parent with youngest child 0–5 vs. 6–15 years				8.311	2.792	0.305 **	6.945	2.771	0.255 *
Social support							−0.188	0.076	−0.234 *
R^2^	0.265			0.330			0.373		
Adjusted R^2^	0.241			0.301			0.339		
R^2^ Change	0.265			0.065			0.043		
F for R^2^ change	11.068 ***			8.859 **			6.188 *		

* *p* < 0.05; ** *p* < 0.01; *** *p* < 0.001; ^a^ Disability (0 = no disability or health condition, 1 = has disability or health condition); ^b^ Employment status (0 = not employed, 1 = Employed (full- or part-time).

## Data Availability

The data that support the findings of this study are available from the corresponding author (FT) upon reasonable request.
